# Radiation-Induced Alterations of Osteogenic and Chondrogenic Differentiation of Human Mesenchymal Stem Cells

**DOI:** 10.1371/journal.pone.0119334

**Published:** 2015-04-02

**Authors:** Séverine Cruet-Hennequart, Carole Drougard, Georgina Shaw, Florence Legendre, Magali Demoor, Frank Barry, Jean-Louis Lefaix, Philippe Galéra

**Affiliations:** 1 Normandy University, Caen, France; UNICAEN, Laboratoire Microenvironnement Cellulaire et Pathologies (MILPAT), Caen, France; 2 Laboratoire Accueil en Radiobiologie avec les Ions Accélérés (CEA-DSV-IRCM-LARIA), Bd Becquerel, Caen Cedex 5, France; 3 Regenerative Medicine Institute (REMEDI), National University of Ireland Galway, Galway, Ireland; Tufts University, UNITED STATES

## Abstract

While human mesenchymal stem cells (hMSCs), either in the bone marrow or in tumour microenvironment could be targeted by radiotherapy, their response is poorly understood. The oxic effects on radiosensitivity, cell cycle progression are largely unknown, and the radiation effects on hMSCs differentiation capacities remained unexplored. Here we analysed hMSCs viability and cell cycle progression in 21% O_2_ and 3% O_2_ conditions after medical X-rays irradiation. Differentiation towards osteogenesis and chondrogenesis after irradiation was evaluated through an analysis of differentiation specific genes. Finally, a 3D culture model in hypoxia was used to evaluate chondrogenesis in conditions mimicking the natural hMSCs microenvironment. The hMSCs radiosensitivity was not affected by O_2_ tension. A decreased number of cells in S phase and an increase in G2/M were observed in both O_2_ tensions after 16 hours but hMSCs released from the G2/M arrest and proliferated at day 7. Osteogenesis was increased after irradiation with an enhancement of mRNA expression of specific osteogenic genes (alkaline phosphatase, osteopontin). Osteoblastic differentiation was altered since matrix deposition was impaired with a decreased expression of collagen I, probably through an increase of its degradation by MMP-3. After induction in monolayers, chondrogenesis was altered after irradiation with an increase in *COL1A1* and a decrease in both *SOX9* and *ACAN* mRNA expression. After induction in a 3D culture in hypoxia, chondrogenesis was altered after irradiation with a decrease in *COL2A1*, *ACAN* and *SOX9* mRNA amounts associated with a *RUNX2* increase. Together with collagens I and II proteins decrease, associated to a *MMP-13* expression increase, these data show a radiation-induced impairment of chondrogenesis. Finally, a radiation-induced impairment of both osteogenesis and chondrogenesis was characterised by a matrix composition alteration, through inhibition of synthesis and/or increased degradation. Alteration of osteogenesis and chondrogenesis in hMSCs could potentially explain bone/joints defects observed after radiotherapy.

## Introduction

Human mesenchymal stem cells (hMSCs) represent a particular stem cell niche in the stromal compartment of the bone marrow. They consist of those stem cells that can differentiate into cells of mesenchymal tissues, including osteoblasts, adipocytes and chondrocytes. hMSCs play an important role in the maintenance of hematopoietic stem cell functions in the bone marrow stromal compartment [1,2]. Furthermore, hMSCs localise to solid tumours, and can modulate cancer cell function through secretion of paracrine signals. While hMSCs, either in the bone marrow, or in the microenvironment of a tumour, will be targeted by DNA damaging agents used in cancer therapy, the response of the hMSC population to irradiation is not well understood [3].

Radiation therapy (RT) using X-rays is a mainstream treatment for many cancers. Despite efficacy in a large number of tumours, radiotherapy has been proven less efficient for radioresistant tumours, including chondrosarcoma [4], and complications arising from radiation therapy are well known [5,6]. Manifestation of radiation toxicity in humans may depend to a large extent on human stem cells, such as mesenchymal stem cells, in the corresponding tissues, and it is recognized that these responses are the major limiting factor in disruption of tissue homeostasis after therapeutic exposure [7]. Osteosarcoma induction after exposure to high Linear Energy Transfer (LET) radiation has been reported [8], linking radiation exposure to cancer of mesenchymal origin. Complications of conventional RT on bone and cartilage in children, including growth dissymmetry, are recognised [9,10]. This bone loss may involve specific endocrine deregulation but also direct damage to the bone marrow osteoprogenitors, thus justifying the interest in characterising the radiation-induced effects on their progenitors.

Unlike the so-called radiosensitive tissues, for which early and late radiation effects have been characterised at the cellular and molecular level, very few studies have investigated the effects of radiation on hMSCs [11,12,13,14,15,16]. In general hMSCs were found to be relatively resistant to irradiation [3,17]. Cells respond to irradiation by activating the DNA damage response (DDR) which can lead to repair of the damage, cell cycle arrest, and activation or inhibition of a specific gene transcription program and cell death [18]. We [13] and others [11,19,20] have found that hMSCs avoid radiation-induced cell death through activation of the DDR pathway leading to cell cycle arrest and DNA damage repair. However, the influence of the microenvironment has been neglected in these previous few studies dealing with the effects of conventional RT on hMSCs. More specifically, oxygen tension is one well known parameter influencing cellular radiosensitivity [21,22]. While hMSCs usually reside *in vivo* in a niche where oxygen tension is relatively low, between 1 and 8% [23,24], most of the studies have been conducted using proliferating hMSCs grown in normoxia conditions (21% O_2_), which do not reflect physiological conditions.

Moreover, data on the radiation-effects on hMSC differentiation capacities has not been obtained in conditions mimicking the microenvironment of hMSCs [15,16], is very limited and still remain controversial. Alterations in the process of differentiation may be detrimental to the balance within the bone marrow and for the mobilisation of hMSCs in response to damage, and may participate to cancer induction [8]. Given this, it is important to consider the effects of radiation on the process of differentiation of these cells in order to define the medium and long term effects of irradiation on hMSC fate and function.

In this study, we examined the effects of medical X-rays on the fate of hMSCs, as defined as their survival and their progression into the cell cycle, in normoxia (21% O_2_) and in physioxia (3% O_2_) conditions. Differentiation towards the osteogenic and the chondrogenic lineages was also induced after 6 Gy and 10 Gy irradiation and studied after optimal differentiation *ie* 21 days and 14 days respectively, both at the cellular levels by specific staining but also at the molecular levels through detailed analysis of differentiation specific gene expression. In order to integrate the microenvironment in the responses of hMSCs to irradiation, chondrogenesis was specifically induced in a 3D model and physioxia conditions after irradiation, to assess the effect of ionising radiation on chondrogenic differentiation.

## Material and Methods

### Cell isolation and X-rays irradiation

Bone marrow aspirates were obtained, with appropriate ethical approval, from normal donors (male; age range: 18 to 25), and hMSCs were isolated and characterized as previously described [13]. Prior to the X-rays irradiations, hMSCs grown in α-MEM supplemented with 10% FBS, 2 mM Glutamine, 100 U/ml penicillin, 0.1 mg/ml streptomycin, 2.5 μg/ml fungizone, and 1 ng/ml FGF-2, were passaged to a maximum of five times. At that concentration, fungizone did not induce DNA double strand breaks (DSBs) as seen by the absence of a marker of DNA strand breaks, the phosphorylated form of histone H2AX (γ-H2AX) [13,25], 1h following mock-irradiation (0 Gy, S1 Fig). S1 Fig also confirms specific induction of DNA DSBs as detected by the presence of the phosphorylated form of histone H2AX (γ-H2AX), 1h after irradiation at 10 Gy [13,25].

Briefly 7500 cells/cm^2^ were seeded, onto standard tissue culture treated polystyrene, in regular culture medium 48h before irradiation. Medium was changed 24h later, and cells were irradiated 24h later. X-rays irradiation was performed at the Comprehensive Care Centre François Baclesse (Caen, France) using a 15MeV, 6mA medical linear accelerator (Saturne 15, Siemens) with a dose rate of 2Gy/min. The same conditions were used for the mock-irradiated cells (0 Gy).

The chondrosarcoma cell line SW1353, purchased from ATCC (ATCC HTB94), was grown in DMEM High Glucose supplemented with 10% FBS, 10 μM ciprofloxacin and 0.5 μg/ml fungizone.

Human articular chondrocytes (HAC), obtained with appropriate ethical approval, were prepared from macroscopically healthy zones of femoral heads of patients undergoing joint arthroplasty, as previously described [26,27].

For experiments in normoxia, cells were grown at 37°C in a humidified atmosphere (21% O_2_) containing 5% CO_2_. For experiments in physioxia, cells were grown in a humidified 37°C hypoxic chamber where O_2_ level was maintained at 3%, with 5% CO_2_ and 94% N_2_.

### Cellular radiotoxicity

Cells were seeded and grown either in normoxia or in physioxia 48h before irradiation. Sixteen hours after irradiation, to allow DSB repair [13,28] as detected by the absence of γ-H2AX 16h after irradiation (S1 Fig), cells were trypsinised and subcultured into 96 well plates at 7500 cells/cm^2^ under standard culture conditions, either in normoxia (21% O_2_) or in physioxia (3% O_2_) for 14 days, day at which XTT test was conducted. Direct radiotoxicity analysis has been performed using the XTT colorimetric test according to the manufacturer’s instruction (Roche Diagnostics). This test determines effects on proliferation and cell viability by measuring the conversion of a yellow tetrazolium salt into an orange formazan dye which only occurs in viable cells. This test has been performed as an alternative to the conventional clonogenicity test [29], since hMSCs do not form colonies [11].

### Cell cycle analysis

Cells were seeded and grown either in normoxia or in physioxia 48h before irradiation. Detailed cell cycle analysis was performed at the time of irradiation (T0-48h culture), and both shortly (16h) and late (7 days) after irradiation. At the indicated times following irradiation, cell cycle progression was analysed by flow cytometry using a Beckman Coulter Gallios flow cytometer (Federative Research Structure ICORE platform, University of Caen/Lower-Normandy-France). Briefly, trypsinised cells were fixed in 70% ethanol and stored at -20°C until staining, for 30 min at 37°C with a PBS solution containing 20 μg/ml propidium iodide and 100 μg/ml RNAse. Data was analysed using Kaluza software. Linear gates have been used to define each subpopulation (G0/G1, S and G2/M), as presented in Fig 2. The subG1 population (representative of the apoptotic population, (less than 5%), and any post G2/M subpopulation (representative of endoreplication, less than 5%), have been gated out, in order to get solely the percentages of the cycling population in each subpopulation (G0/G1, S and G2/M).

### Methylene blue and senescence staining

At the indicated times (7 days, 14 days and 21 days) following irradiation with 6 Gy and 10 Gy, and/or differentiation, cells were fixed and stained for 45 min using 0.25% methylene blue in 50% ethanol. Photographs were taken using a Zeiss microscope at a 10X objective.

For senescence detection 7 days after irradiation, the senescence β-Galactosidase staining kit was used according to manufacturer’s instructions (Cell Signaling). Photographs were taken using a Zeiss microscope at a 10X objective.

### Differentiation induction and analysis

In order to directly address the effect of irradiation on the abilty of hMSCs to differentiate into osteoblasts and chondrocytes, osteogenic and chondrogenic differentiation were induced following irradiation, using recognised protocols for osteoblastic or chondrogenic differentiation [26,27,30,31,32]. Cells were seeded and irradiated as described in the “cell isolation and X-rays irradiation” section. At the time of differentiation induction, the confluency was 80%.

**Fig 1:**
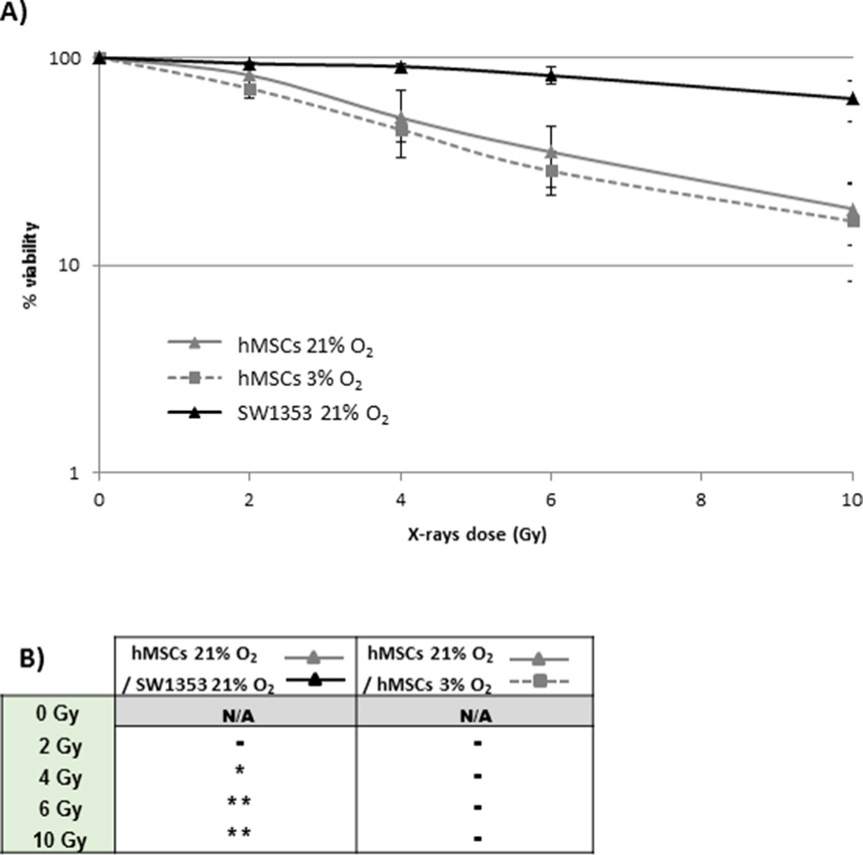
Viability of hMSCs following X-rays irradiation, under normoxia (21% O_2_) and physioxia (3% O_2_) growing conditions. A) hMSCs were grown in normoxia (21% O_2_) or physioxia (3% O_2_) for 48h before X-rays irradiation, and 14 days following irradiation. Cells irradiated at the indicated doses of X-rays were trypsinised and seeded onto 96 wells plates, 16 after irradiation. Cell viability was determined using the XTT assay 14 days after reseeding, and is expressed as a percentage of the viability of untreated cells. Data represent the mean of five independent experiments ± SD using hMSCs from three different donors in normoxia conditions, and the mean of four independent experiments ± SD using hMSCs from three different donors in physioxia conditions. For the SW1353 cells, data represent the mean of four independent experiments ± SD. B) Statistical analysis was performed by unpaired Student t test (* p<0.05, ** p<0.01). N/A: not available.

Osteogenic and chondrogenic differentiation were induced 16h after irradiation at 6 Gy, which corresponds to the D_37%_ as described in Fig 1, and after 10 Gy which is near the D_10%_ value and corresponds to the maximal dose delivered

**Fig 2:**
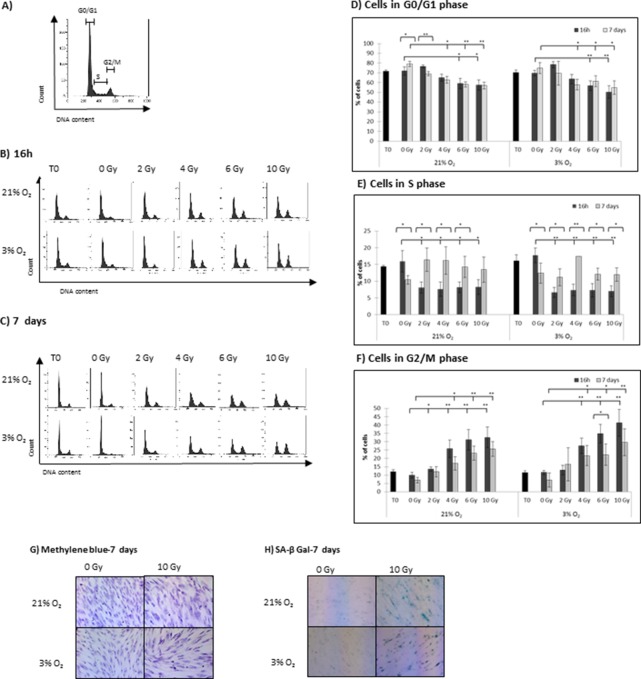
Cell cycle progression of hMSCs following X-rays irradiation, under normoxia (21% O2) and physioxia (3% O2) growing conditions. A) Representative histogram, showing the cell cyce phases of hMSCs grown in normoxia for 48h. B-C) hMSCs grown in normoxia (21% O2) or physioxia (3% O2) for 48h before X-rays irradiation, and for the indicated times after irradiation, were harvested and fixed as described in the Material and Methods. DNA was stained with propidium iodide (PI), and DNA content was analyzed by flow cytometry for the determination of cell cycle distribution. Histograms from a representative experiment 16h (A), and 7 days (B) following irradiation are presented. D-E-F) Percentages of cells in the different phases of the cell cycle (as determined using flow cytometry histograms like in A) are presented. Data represent the mean of four independent experiments ± SEM using hMSCs from three different donors for the 16h time point, and up to 9 experiments using hMSCs from five different donors for the 7 day time point. Statistical analysis was performed by unpaired Student t test (* p<0.05, ** p<0.01). G) hMSCs grown in normoxia (21% O2) or physioxia (3% O2) 48h before X-rays irradiation, and for 7 days following irradiation, were fixed and stained using methylene blue. Pictures (Objectives X10) from a representative experiment (n = 4 in normoxia, n = 2 in physioxia) 7 days following irradiation are presented. H) hMSCs grown in normoxia (21% O2) or physioxia (3% O2) 48h before X-rays irradiation, and for 7 days following irradiation, were fixed and stained using the senescence-β Galactosidase kit. Pictures (Objectives X10) from a representative experiment (n = 2) 7 days following irradiation are presented.

Differentiation was assessed both at the cellular level and at the molecular level after 21 days for osteogenic differentiation and 14 days for chondrogenic differentiation, which correspond to optimal differentiation conditions [26,27,30,31,32]. Results were obtained using hMSCs isolated from the bone marrow of four healthy donors.

#### Induction of osteoblast differentiation

Osteogenic differentiation (defined as OS in figures) was induced in monolayer cultures using regular culture medium (α-MEM 10% FBS, 2 mM Glutamine, 1 ng/ml FGF-2, 1% penicillin/streptomycin) supplemented with osteogenic factors (100 nM Dexamethasone, 10 mM β-glycerophosphate, 50 μM ascorbate 2-phosphate) [31,33]. Medium was changed every 7 days up to 21 days.

#### Analysis of osteoblast differentiation

21 days following induction of differentiation, cells were rinsed in PBS, fixed with 4% PFA for 15 min, and stained with 2% Alizarin red solution (pH4.1) for 20 minutes. Alizarin red solution detects calcium deposits. Mineralization can also be visualised by dense, refractile deposits using light microscopy [33]. Photographs were taken using a Zeiss microscope and a 10X objective.

Cells were also harvested after 21 days of differentiation for RNA extraction and real time RT-PCR analysis, in order to analyse the mRNA expression levels of osteogenic-related markers, such as alkaline phosphatase (*ALP*), osteopontin (*OPN*) and bone sialoprotein (*BSP*) and transcription factors genes, such as Runx2 (*RUNX2*) [34,35,36], as well as the mRNA expression level of *SOX9*, a chondrogenic transcription factor inhibiting osteogenesis [37].

Since osteoblasts are known to deposit mostly a collagenous matrix [36,38], cells were also harvested after 21 days of differentiation for protein extraction and western blot analysis to follow the expression of type I, and type II collagen.

#### Induction of chondrogenic differentiation in monolayer culture

Chondrogenic differentiation (defined as CH-2D in figures) was induced in monolayer cultures using ICM (*Incomplete Chondrogenic Medium*) supplemented with two known inducers of chondrogenesis, BMP-2 (50 ng/ml), and TGF-β1 (10 ng/ml) [39,40]. ICM medium consists of DMEM 4.5 g/ml glucose, 1 mM sodium pyruvate, 100 nM dexamethasone, 50 μg/ml ascorbic acid-2-phosphate, 40 μg/ml proline, and Insulin Transferin Selenium. ICM was used as medium for undifferentiated cells (defined as ND in figure legends). Cultures were maintained in a normoxic environment for 14 days and medium (ICM or ICM + BMP-2 + TGF-β1) was changed every 3 days for the duration of the experiment.

#### Analysis of chondrogenic differentiation in monolayer culture

14 days after differentiation induction in monolayer cultures, cells were rinsed in PBS, fixed with 4% PFA for 15 min, and stained using 1% alcian blue solution (pH1) for 30 min. Alcian blue staining allows detection of sulfated polysaccharides. Photographs were taken using a Zeiss microscope at a 10X objective.

Cells were also harvested after 14 days of differentiation for RNA extraction and real time RT-PCR analysis, in order to analyse the mRNA expression levels of specific makers of chondrogenesis, including *COL2A1*, *ACAN*, and *SOX9*, and that of dedifferentiated and hypertrophic chondrocytes, including *COL1A1*, *RUNX2*, and *COL10A1* [26,40,41].

#### Induction of chondrogenic differentiation in 3D culture and physioxia

hMSCs were grown for 14 days in 3D scaffold to induce chondrogenesis [32] according to the following protocol. Briefly, cells were trypsinised 16h following irradiation and 500 000 cells were seeded into 3D scaffolds manufactured by Symatèse Biomatériaux (Chaponost, France) as previously described [26,32]. These collagen sponges (2 mm thickness, 5 mm diameter), are composed of native type I collagen (90–95%) and type III collagen (5–10%) from calf skin, crosslinked using glutaraldehyde to increase their stability and sterilized with ß-radiation.

hMSCs were seeded onto the scaffolds in 96-well culture plates and incubated at 37°C and 5% CO_2_ for 1h. Scaffolds were transferred into 24-well plate and media with or without 50 ng/ml of BMP-2 (R&D Systems) plus 10 ng/ml of TGF-β1 (R&D system) was added. Undifferentiated controls (ND: not differentiated) were grown using ICM without BMP-2 nor TGF-β1, while differentiation (CH-3D: induced chondrogenesis) was induced using ICM supplemented with BMP-2 and TGF-β1 (as for culture in monolayer). Cells in scaffolds were then incubated in physioxia (3% O_2_) for 14 days. This point established the day zero point. Medium was changed on days 4, 7, 11 and sponges were harvested for RNA and proteins on day 14. Each set of samples (± BMP-2, TGF-β1) had the same medium replacement protocol.

#### Analysis of chondrogenic differentiation in 3D culture and physioxia

Cells in scaffolds were harvested after 14 days of differentiation for RNA extraction and real-time RT-PCR analysis, in order to analyse the mRNA expression levels of specific makers of chondrogenesis, including *COL2A1*, *ACAN*, and *SOX9*, and that of dedifferentiated and hypertrophic chondrocytes, including *COL1A1*, *RUNX2*, and *COL10A1* [26,40,41]. Cells were also harvested after 14 days of differentiation for protein extraction and Western-blot analysis to follow the expression of type I and type II collagens.

### RT-PCR Analysis

The day of induction (T0), 14 days after chondrogenesis induction and 21 days after osteogenic induction, cells in monolayers or in scaffolds were harvested for RNA extraction and real time RT-PCR analysis. Total RNAs were extracted using TRIzol reagent, and reverse transcribed into complementary DNA using MMLV before amplifications), using the primers described below and SYBR Green Real-Time PCR Master Mix, in a real-time PCR machine (Applied Biosystems Step one Plus) as previously described [27]. Ribosomal protein L13a (*RPL13A*) was used as endogenous reference gene. The relative gene expression was calculated with the 2-^ΔΔCt^ method between *RPL13A* and each target gene. Samples were run as triplicates and results of a minimum of four experiments were plotted as box and whisker plots, with minimum and maximum, median and mean plotted.

Quantitative real time reverse transcription polymerase chain reaction (qRT-PCR) has been used to analyse specific differentiation markers of osteogenesis and chondrogenesis.

The following primer sequences were used:

*RPL13A*  GAG GTA TGC TGC CCC ACA AA  GTG GGA TGC CGT CAA ACA C

*COL2A1*  GGC AAT AGC AGG TTC ACG TAC A  CGA TAA CAG TCT TGC CCC ACT T

*COL1A1*  CAC CAA TCA CCT GCG TAC AGA A  CAG ATC ACG TCA TCG CAC AAC

*COL10A1*  AAA CCA GGA GAG AGA GGA CCA TAT G  CAG CCG GTC CAG GGA TTC

*SOX9*  CCC ATG TGG AAG GCA GAT G  TTC TGA GAG GCA CAG GTG ACA

*RUNX2*  GCA GCA CGC TAT TAA ATC CAA ATT  ACA GAT TCA TCC ATT CTG CCA CTA G

*ALP*  AGC CCA GAG ATG CAA TCG  CTA TCC TGG CTC CGT GCT C

*OPN_OSTEOPONTIN*  CTC AGG CCA GTT GCA GCC  CAA AAG CAA ATC ACT GCA ATT CTC

*OCN_OSTEOCALCIN*  CGG TGC AGA GTC CAG CAA A  GGT AGC GCC TGG GTC TCT TC

*BSP*  TGC CTT GAG CCT GCT TCC  GCA AAA TTA AAG CAG TCT TCA TTT TG

*MMP-1*  GAA GCT GCT TAC GAA TTT GCCG  CCA AAG GAG CTG TAG ATG TCC T

*MMP-3*  TAA AGA CAG GCA CTT TTG GCG C  TTG GGT ATC CAG CTC GTA CCT C

*MMP-13*  AAG GAG CAT GGC GAC TTC T  TGG CCC AGG AGG AAA AGC

### Western-blotting

One hour and sixteen hours after irradiation, the day of induction (T0), 14 days after chondrogenesis induction and 21 days after osteogenic induction, cells in monolayers or in scaffolds were washed in PBS, and lysed in RIPA buffer (NaCl 150 mM, 50 mM Tris-HCl pH7.4, 1% NP40, 0.25% DOC, 1 mM EDTA) containing 1 mM Na_3_VO_4_, 5 mM NaF, 1 mM PMSF, 2 μg/ml aprotinin and 1 μg/ml leupeptin.

Protein concentration was determined using the Bradford protein assay (BioRad). Proteins were separated by 10% SDS-PAGE, and transferred to PVDF membrane. Membranes were blocked for 1h in T-TBS Milk 10% and incubated overnight at 4°C with the following antibodies in T-TBS Milk 1%: anti-H2AX phosphoserine 139 (γ-H2AX, Millipore, 1/1000), anti-p53 (Santa Cruz, 1/1000), anti-type I collagen (Sigma, 1/3000), anti-type II collagen (Novotec 1/1000), and anti-GAPDH (Santa Cruz, 1/7500) as a loading control. After T-TBS washes, membranes were incubated for 1h at room temperature with anti-rabbit or anti-mouse HRP-coupled secondary antibodies (Jackson Immunochemicals) for 1h at room temperature in T-TBS Milk 3%. Bound antibody was detected using the ECL chemiluminescence method (Pierce).

### Statistical analysis

GraphPad Prism was used for statistical analysis, and statistical analysis is detailed in the legend of each figure.

### Ethics Statement

The ethical issue involved in this research is the use of human biological samples in the form of primary human mesenchymal stem cells (hMSCs). hMSCs are adult stem cells isolated from bone marrow aspirates from healthy donors in the Regenerative Medicine Institute [REMEDI] at the National University of Ireland Galway (http://www.remedi.ie/people/prof-frank-barry). REMEDI has many years of experience in obtaining these cells and holds the relevant Informed consent forms according to the NUIGalway Research Ethics committee’s guidelines. It is the objective of the NUIG Galway Research Ethics Committee (REC) to safe-guard the health, welfare and rights of human participants in research studies and to afford dignity to the handling and treatment of biological material, taking into account the concerns of the local community.

At NUI Galway, the University College Hospital Ethics Committee currently grants ethical approval for research on human subjects or their tissues. This committee has been in operation for a number of years. From early-2005, a University Research Ethics Committee will grant ethical approval for research on human subjects or their tissues, not including clinical trials work, which will remain the responsibility of the Hospital Ethics Committee. Both committees comply with the guidelines laid out in the Operational Guidelines for Ethics Committees that Review Biomedical Research (2000), World Health Organisation, Geneva (TDR/PRD/ETHICS/2000.1), Guidelines and Recommendations for European Ethics Committees (1997). European Forum for Good Clinical Practice and the EU Directive on Clinical Trials (Directive 2001/20/EC), which were transposed in Irish law in May 2004 (European Communities (Clinical Trials on Medicinal Products for Human Use) Regulations 2004) and most recently the guidelines of the Irish Council for Bioethics. Marrow samples used in the present study were taken from volunteer donors with informed consent and according to a protocol approved by Galway University Hospital Ethics Committee. All aspects of the research have been approved by the Institutional Ethics Review Board. All identifying information of the human biological samples was removed before the authors received them. The informed consent forms that REMEDI received for collection of these stem cells covers storage of the samples and possible use in future research.

A second ethical issue involved in this research is the use of human biological samples in the form of primary human articular chondrocytes (HAC). HAC were prepared from macroscopically healthy zones of femoral heads of patients undergoing joint arthroplasty at the Dept. of Surgery of St Martin Clinic (Caen, France). Our group has numerous years of experience using this cell type [26, 27, 32]. All patients signed an informed consent agreement form, which was approved by the local Ethics Committee for research with human samples (Comité de Protection des Personnes Nord Ouest III).

## Results

### MSCs survive X-irradiation in normoxia and in physioxia

The sensitivity of hMSCs grown in physioxia (3% O_2_) was compared to that of hMSCs grown in normoxia (21% O_2_). As seen in Fig 1A, hMSCs grown in normoxia were more sensitive to irradiation than the SW1353 cell line when grown in the same conditions (compare the two full lines). This difference in sensitivity was statistically significant from 4 Gy up to 10 Gy, as seen in the statistical analysis presented in Fig 1B (hMSCs 21% O_2_ / SW1353).

For both hMSCs grown in normoxia and physioxia, viability decreased in a dose-dependent manner after irradiation (Fig 1A), and no significant difference was observed between hMSCs grown in physioxia and hMSCs grown in normoxia (Fig 1B).

Though more sensitive than the chondrosarcoma cells, hMSCs have a D_37%_ value near 6 Gy (irradiation dose corresponding to a surviving fraction of around 37%) and a D_10%_ value (irradiation dose corresponding to a surviving fraction of around 10%) near 10 Gy. These two doses were chosen for subsequent experiments, since both D_10%_ and D_37%_ are commonly used in radiobiology [42,43].

### MSCs are blocked in G2/M after irradiation, both in normoxia and in physioxia, but are able to resume proliferation

Detailed cell cycle analysis was performed at the time of irradiation (T0), and both early (16h) and late (7days) after irradiation. As seen in the cell cycle profiles (Fig 2A, 2B and 2C) and the histograms (Fig 2D, 2E and 2F), 70% of hMSCs are in G0/G1 phase both in normoxia and physioxia, and no significant difference was observed between the two conditions. After either 16h or 7 days, no significant difference in the cell cycle distribution was observed between cells grown in normoxia and in physioxia after irradiation (data not shown). However, in both conditions, a decrease in the number of hMSCs in S phase and an increase of hMSCs in the G2/M phase of the cell cycle were observed 16h after irradiation at 2 Gy. From 4 Gy to 10 Gy, an increase of cells in G2/M was observed together with a low number of cells in S phase (<7%) and a decreased number of cells in G0/G1, 16h after irradiation. Seven days after irradiation, an increase in the number of cells in S phase was observed compared to the number of cells in S phase 16h after irradiation. This increase in S phase was accompanied by a decrease in the number of cells in G2/M, and indicative of proliferation.

Data presented here show that cell cycle progression was influenced by X-irradiation, with a decreased number of cells in S phase and a remarkable increase of cells in G2/M indicative of a radiation-induced G2/M arrest, both in normoxia and in physioxia 16h after irradiation. However, 7 days after irradiation, more hMSCs were in S phase and progressed through the cell cycle, both in normoxia and in physioxia. Is it worth noting that the cell cycle still has not returned to ‘normal’ even by 7 days after irradiation.

As seen in the methylene blue photographs (Fig 2G), cellular morphology was also influenced by X-irradiation, with cells becoming enlarged and flattened 7 days after irradiation, both in normoxia and in physioxia. A lower density was also observed in accordance with the cell viability results (Fig 1). Moreover, while the presence of senescent cells after 10 Gy was observed (Fig 2H), no morphological signs of apoptosis were detected (Fig 2G), nor was an increase in the subG1 population observed by cell cycle analysis (data not shown), both in normoxia and in physioxia. These data indicate that hMSCs rather undergo radiation-induced senescence rather than radiation-induced apoptosis.

### Osteogenic differentiation of MSCs is altered after X-ray irradiation

**Fig 3:**
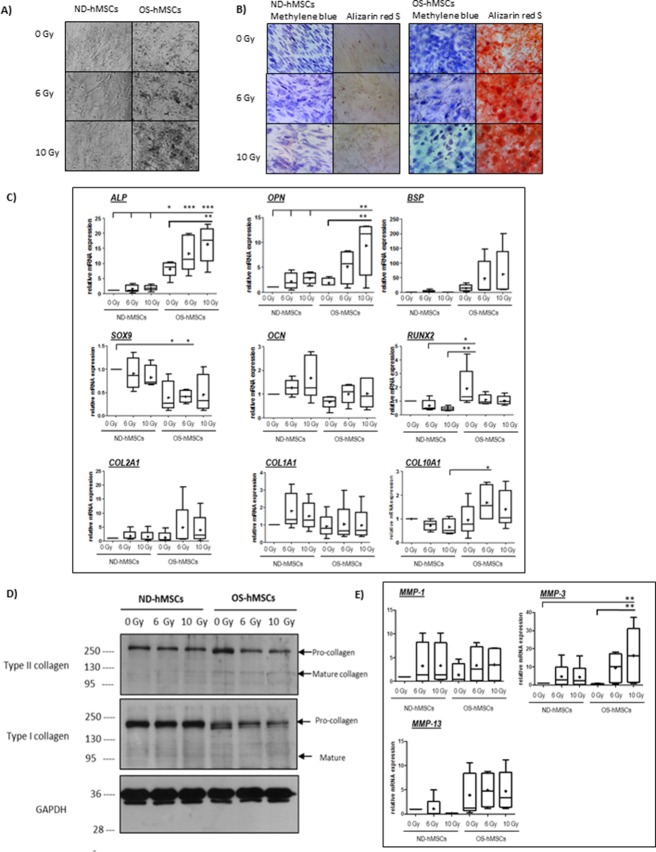
Effect of X-irradiation on osteogenic differentiation of hMSCs. hMSCs were mock-irradiated or irradiated at 6 Gy and 10 Gy. 16h following X-rays irradiation, osteogenic differentiation (OS-hMSCs) was induced and maintained using osteogenic differentiation medium for 21 days as described in Material and Methods. Undifferentiated hMSCs (ND-hMSCs) were grown in regular culture medium for 21 days. A) Light microscopy photographs of hMSCs grown for 21 days in complete regular culture media (ND-hMSCs) or osteogenic differentiation media (OS-hMSCs). Photographs are representative of data from (Objectives X10) 3 donors. B) Photographs of hMSCs after methylene blue and alizarin red S staining, 21 days following osteogenic differentiation after irradiation (0-6-10 Gy). Methylene blue staining was used to assess hMSCs morphology in undifferentiated (ND-hMSCs) and osteogenically-differentiated cells (OS-hMSCs) after irradiation. Alizarin red S staining was used to detect the presence of calcium deposition in undifferentiated (ND-hMSCs) and osteogenically- differentiated cells (OS-hMSCs) after X-rays irradiation. Representative photographs (Objectives X10) of 3 donors. C) Real-time RT-PCR analysis of relative mRNA expression of the indicated genes (*ALP*-alkaline phosphatase, OPN-osteopontin, BSP-Bone sialoprotein, SOX9-Sox9 transcription factor, *OCN*-Osteocalcin, *RUNX2*-runt-related transcription factor 2, *COL1A1*-type I collagen, *COL10A1*-type X collagen, *COL2A1*-type II collagen) in undifferentiated hMSCs (ND-hMSCs) and osteogenically differentiated hMSCs (OS-hMSCs) after irradiation (0-6-10 Gy). Gene expression is normalized against the endogenous reference gene, *RPL13A*. Results are expressed as relative mRNA expression as compared to the control condition (undifferentiated hMSCs, 0 Gy condition) set at the arbitrary unit of 1 Box and whiskers plots (Min to Max. Bar at median and + at mean) of data derived from 5 donors. Statistical analysis (One-Way ANOVA, with Tukey post-test) was performed and statistical significance is shown with, * for p<0.05, and ** for p< 0.01. D) Western blot analysis of type II collagen, type I collagen, and GAPDH in undifferentiated (ND-hMSCs) and osteogenically differentiated cells (OS-hMSCs) after irradiation. E) Real-time RT-PCR analysis of expression levels of the indicated genes (*MMP-1*, *MMP-3*, and *MMP-13*) in undifferentiated hMSCs (ND-hMSCs) and osteogenically differentiated hMSCs (CH 3D-hMSCs) after irradiation (0-6-10 Gy). Gene expression is normalized against the endogenous reference gene, *RPL13A*. Results are expressed as relative mRNA expression as compared to the control condition (undifferentiated hMSCs, 0 Gy condition) set at the arbitrary unit of 1. Box and whiskers (Min to Max. Bar at median and + at mean) of 4 independent experiments. Statistical analysis (One-Way ANOVA, with Tukey post-test) were performed and statistical significance is shown with, * for p<0.05, and ** for p< 0.01.

Osteogenic differentiation was induced after X-irradiation and assessed 21 days later. As seen in light microscopy photographs (Fig 3A), dense, refractile deposits, indicative of mineralization were seen in osteogenically differentiated hMSCs but not in the undifferentiated hMSCs (compare ND-hMSCs and OS-hMSCs at 0 Gy). In addition, a dose-dependent increase in the density of these deposits was observed in the irradiated and osteogenically differentiated hMSCs (OS-hMSCs), with denser deposits being observed after 10 Gy compared to 6 Gy. In order to specifically assess that these deposits were genuine calcium deposits, we performed an Alizarin red solution staining. Methylene blue staining was also performed in order to show the morphological changes associated with osteoblastic differentiation. As seen in Fig 3B (methylene blue), osteogenically differentiated hMSCs (OS-hMSCs) presented a more cuboidal morphology, characteristic of osteoblasts than the undifferentiated hMSCs, indicating proper differentiation induction. Moreover, no alizarin red staining was observed in the undifferentiated hMSCs, while osteogenically differentiated hMSCs exhibited a strong red staining indicative of calcium deposition and osteogenic differentiation. hMSCs, that had been osteogenically differentiated after 6 Gy and 10 Gy, despite showing a lower density than the undifferentiated hMSCS, presented a more pronounced alizarin red staining compared to the mock-irradiated OS-hMSCs, and displayed mineralized nodules, indicative of increased differentiation after 6 and 10 Gy.

In order to directly assess the level of osteogenic differentiation after X-irradiation, we analysed the mRNA expression levels of osteogenic-related markers, such as alkaline phosphatase (*ALP*), osteopontin (*OPN*) and bone sialoprotein (*BSP*) and transcription factors, such as Runx2 (*RUNX2*), in hMSCs grown in regular culture medium compared to hMSCs grown in osteogenic medium (Fig 3C). As expected, osteogenic medium induced OS-hMSCs to display higher relative mRNA expression of the osteogenic markers *ALP*, *BSP* and *RUNX2*, and lower relative mRNA expression of *SOX9* (chondrogenic transcription factor, inhibitor of osteogenesis) compared to ND-hMSCs (compare 0 Gy conditions). The mean relative mRNA expression of *ALP* and *BSP* in differentiated hMSCs (OS-hMSCs) was respectively 8.2, and 16.3 times higher than in non-differentiated hMSCs (ND-hMSCs) (compare 0 Gy conditions). However, due to differences between donors in their intrinsic ability to differentiate into osteoblasts [44], the difference between ND-hMSCs and OS-hMSCs was only significant for *ALP*. When expression of these osteogenic markers was analysed in individual donors, the increase of *ALP*, and *BSP* was statistically significant (between p<0.05 and p<0.001) between the non-differentiated hMSCs (ND-hMSCs) and the osteogenically differentiated hMSCs (OS-hMSCs) (S2 Fig).

In addition, we observed a significant increase in both *ALP* and *OPN*, and a trend towards an increase in *BSP* in osteogenically-differentiated hMSCs after irradiation (Fig 3C). The mean levels of *ALP*, *OPN*, and *BSP* in osteogenically differentiated hMSCs after 10 Gy (OS-hMSCs-10 Gy), were respectively increased by 2 for *ALP*, by 5 for *OPN* and by 4 for *BSP* compared to mock-irradiated osteogenically-differentiated hMSCs (OS-hMSCs-0Gy). Again, due to differences between donors in their intrinsic ability to differentiate into osteoblasts, the difference between mock-irradiated OS-hMSCs and irradiated OS-hMSCs (Fig 3C) was not significant for *BSP*, while it was when individual donors were analysed (S2 Fig). When looking at another osteogenic marker, osteocalcin (*OCN*) no major change was observed, with mean levels being close to 1 across all conditions (Fig 3C).

Analysis of the expression of collagenous extracellular matrix proteins showed that no significant changes in the relative mRNA expression of *COL1A1*, *COL10A1* nor *COL2A1* were observed after osteogenic differentiation (compare 0 Gy ND-hMSCs / 0 Gy OS-hMSCs). After irradiation, no change in *COL1A1* expression was observed. A trend towards increased relative mRNA expression of *COL10A1* and *COL2A1* was observed after irradiation, with the mean relative mRNA expression was going from 1.0 to 1.4 for *COL10A1* and from 1.2 to 3.9 for *COL2A1*, between mock-irradiated OS-hMSCs and OS-hMSCs after 10 Gy.

Altogether these results support an alteration in hMSCs differentiation towards the osteogenic lineage after X-irradiation.

### Collagen expression by osteogenically differentiated hMSCs is impaired after irradiation

Since osteoblasts are known to deposit mostly a collagenous matrix, we decided to look at the expression of collagenous proteins expressed by the differentiated and undifferentiated hMSCs after irradiation by Western-blot. As seen in Fig 3D, the procollagen and, to a lesser extent, the mature form of type II collagen were increased in OS-hSMCs compared to ND-hMSCs (compare 0 Gy conditions). After irradiation, a decrease in the procollagen and mature form of type II collagen was observed. Expression of type I collagen was also modified after osteogenesis induction, as seen by a change in the procollagen band pattern. A decrease in the expression of the type I procollagen was observed in the irradiated OS-hMSCs, with no change in the level of the mature form. These data suggest that matrix deposition occurring during osteogenesis is altered in irradiated OS-hMSCs. The decreased expression of type II collagen (Fig 3D) was associated with an increase in *MMP-3* mRNA (Fig 3E). However, due to differences between donors in their intrinsic ability to differentiate into osteoblasts [44], the difference between ND-hMSCs and OS-hMSCs was only significant for *MMP-3*. When expression was analysed in individual donors, the increase of *MMP-3*, and *MMP-13* was statistically significant between the non-differentiated hMSCs (ND-hMSCs) and the osteogenically differentiated hMSCs (OS-hMSCs) (S3 Fig). The increase in *MMP-3* and *MMP-13* could contribute to the decreased expression of type II and type I collagens after X-irradiation.

### Chondrogenic differentiation of MSCs is altered after irradiation

**Fig 4:**
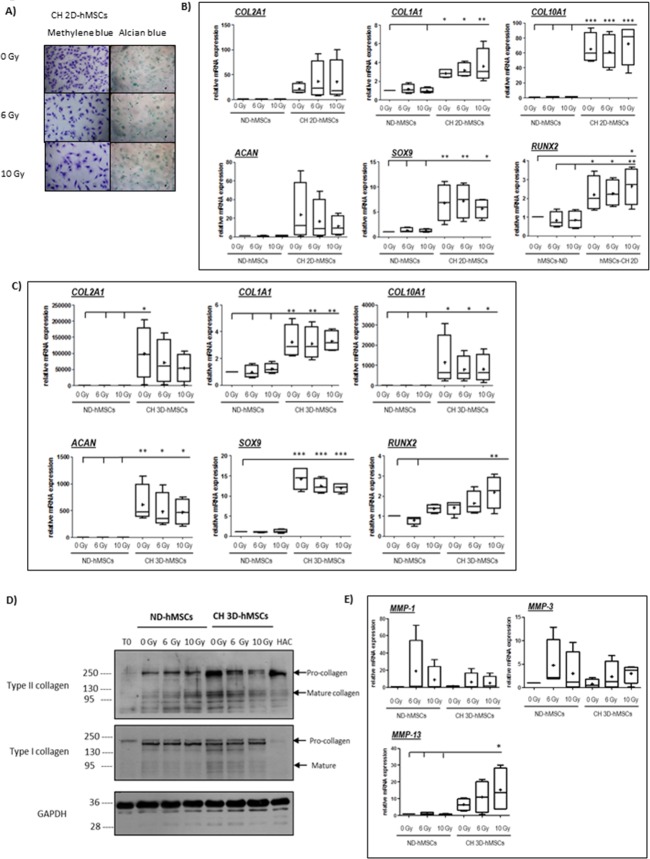
Effect of X-irradiation on chondrogenic differentiation of hMSCs. A-B) hMSCs were mock-irradiated or irradiated with the indicated doses (0-6-10 Gy). 16h following irradiation, cells were seeded in 6 wells plates (2D culture) and chondrogenic differentiation (CH 2D-hMSCs) was induced and maintained using chondrogenic differentiation medium for 14 days in normoxia as described in Material and Methods. Undifferentiated hMSCs (ND-hMSCs) were grown in regular culture medium for 14 days. A) Photographs of hMSCs after methylene blue and alcian blue staining at pH1, 14 days following chondrogenic differentiation after irradiation (0-6-10 Gy). Methylene blue staining was used to assess hMSCs morphology in chondrogenically differentiated cells (CH 2D-hMSCs) after irradiation. Alcian blue staining was used to detect the presence of sulphated proteoglycans in chondrogenically differentiated cells (CH 2D-hMSCs) after irradiation. Representative photographs (Objectives X10) of 2 donors are shown. B) Real-time RT-PCR analysis of expression levels of the indicated genes (*COL2A1*-type II collagen, *COL1A1*-type I collagen, *COL10A1*-type X collagen, *ACAN*-aggrecan, and *SOX9*-transcription factor, *RUNX2*-runt-related transcription factor 2) relative to *RPL13A*, in undifferentiated hMSCs (ND-hMSCs) and chondrogenically differentiated hMSCs (CH 2D-hMSCs) after irradiation (0-6-10 Gy). Gene expression is normalized against the endogenous reference gene, *RPL13A*. Results are expressed as relative mRNA expression as compared to the control condition (undifferentiated hMSCs, 0 Gy condition) set at the arbitrary unit of 1. Box and whiskers plots (Min to Max. Bar at median and + at mean) of 4 donors (n = 4). Statistical analysis (One-Way ANOVA, with Tukey post-test) was performed and statistical significance is shown with, * for p<0.05, and ** for p< 0.01. C-D-E) hMSCs were mock-irradiated or irradiated with the indicated doses (6–10 Gy). 16h following irradiation, cells were included into type I/III collagen sponges (3D culture) and chondrogenic differentiation (CH 3D-hMSCs) was induced and maintained using chondrogenic differentiation (incomplete chondrogenic medium + BMP-2 + TGF-β1) medium for 14 days in physioxia as described in Material and Methods. Undifferentiated hMSCs (ND-hMSCs) were also grown in type I/III collagen sponges, but in incomplete chondrogenic medium in physioxia for 14 days as described in Material and Methods. C) Real-time RT-PCR analysis of expression levels of the indicated genes (*COL2A1*-type II collagen, *ACAN*-aggrecan, *SOX9*-Sox9 transcription factor, *COL1A1*-type I collagen, *COL10A1*-type X collagen, *RUNX2*-runt-related transcription factor 2) in undifferentiated hMSCs (ND-hMSCs) and chondrogenically-differentiated hMSCs (CH 3D-hMSCs) after irradiation (0-6-10 Gy). Gene expression is normalized against the endogenous reference gene, *RPL13A*. Results are expressed as relative mRNA expression as compared to the control condition (undifferentiated hMSCs, 0 Gy condition) set at the arbitrary unit of 1. Box and whiskers plots (Min to Max. Bar at median and + at mean) of 4 independent experiments. Statistical analysis (One-Way ANOVA, with Tukey post-test) were performed and statistical significance is shown with, * for p<0.05, and ** for p< 0.01. D) Western blot analysis of type II collagen, type I collagen, and GAPDH in undifferentiated (ND-hMSCs) and chondrogenically differentiated cells (CH 3D-hMSCs) after irradiation. Representative of 3 donors. A control of human articular chondrocytes (HAC) is shown. E) Real-time RT-PCR analysis of expression levels of the indicated genes (*MMP-1*, *MMP-3*, and *MMP-13*) in undifferentiated hMSCs (ND-hMSCs) and chondrogenically differentiated hMSCs (CH 3D-hMSCs) after irradiation (0-6-10 Gy). Gene expression is normalized against the endogenous reference gene, *RPL13A*. Results are expressed as relative mRNA expression as compared to the control condition (undifferentiated hMSCs, 0 Gy condition) set at the arbitrary unit of 1. Box and whiskers plots (Min to Max. Bar at median and + at mean) of 4 independent experiments. Statistical analysis (One-Way ANOVA, with Tukey post-test) were performed and statistical significance is shown with, * for p<0.05, and ** for p< 0.01.

In order to analyze the effects of irradiation on chondrogenesis in comparable conditions as for osteogenesis, we then studied the radiation-induced effects on the ability of hMSCs to differentiate into chondrocytes in monolayer culture, although we know that monolayer culture is not optimal for chondrogenesis induction. As seen in Fig 4A, 14 days after incubation with the chondrogenic medium, hMSCs presented a polygonal morphology and stained positive for alcian blue, suggesting differentiation (Fig 4A). When chondrogenesis was induced in hMSCs after irradiation, fewer chondrocytes was seen and these displayed a larger size and a more cuboidal morphology, indicative of reduced proliferation after irradiation and alteration of chondrocytes differentiation.

When looking at specific makers of chondrogenesis (*COL2A1*, *ACAN*, *SOX9*), we observed only a significant increase in the relative mRNA expression of *SOX9*, when hMSCs were grown in the chondrogenic medium (Fig 4B) (ND- versus CH 2D-hMSCs 0 Gy). However, a significant increase in the relative mRNA expression of *COL1A1*, *COL10A1* and *RUNX2*, markers of dedifferentiated and hypertrophic chondrocytes, was also observed, indicating poor chondrogenesis. When looking at the effect of irradiation on chondrogenesis differentiation in these conditions, only trends were observed, with a trend towards an increase in *COL2A1*, and *COL1A* relative mRNA expression, and a decrease in *ACAN* and *SOX9* relative mRNA expression, suggesting alteration of the chondrogenic differentiation ability of hMSCs after irradiation. Since the initial chondrogenic phenotype observed in these conditions was that of dedifferentiated chondrocytes (high *COL1A1* and *COL10A1*), we took advantage of our experience with a human articular chondrocytes redifferentiation model [26,27,32], and applied it to hMSCs (Fig 4C). After irradiation, chondrogenesis was induced in hMSCs grown in 3 dimensions in type I/III collagen sponges, and in physioxia. Much higher relative mRNA expression of *COL2A1* and *ACAN* in differentiated hMSCs was observed in these 3D and physioxia conditions compared to the monolayer and normoxia condition (compare Fig 4B and Fig 4C). The mean relative mRNA expression of *COL2A1* and *ACAN* in the differentiated 3D model in physioxia, were respectively 99190 and 610 in the differentiated hMSCs in the 3D model (Fig 4C), while they were only 23 and 23.8 in the differentiated hMSCs in the monolayer conditions (Fig 4B). A significant increase in *COL2A1*, *ACAN* and *SOX9* mRNA amounts was observed between the non-differentiated hMSCs and differentiated (compare 0 Gy conditions) hMSCs. This, together with highly elevated levels of *COL2A1*, *ACAN* and *SOX9* present these conditions as more appropriate to induce chondrogenesis than the monolayer culture conditions.

Using this 3D and physioxia conditions, we analysed the radiation-induced effects on the chondrogenic differentiation ability of hMSCs. As seen in Fig 4C, a trend towards a dose-dependent decrease in the relative mRNA expression of *COL2A1*, *ACAN*, *SOX9* was observed. Between 0 Gy and 10 Gy, the mean levels of *COL2A1*, *ACAN* and *SOX9* in chondrogenically differentiated hMSCs after irradiation were decreasing from 99190 to 53807 relative mRNA expression for *COL2A1*, 610 to 469 relative mRNA expression for *ACAN* and 14.2 to 12 relative mRNA expression for *SOX9*. Due to the differences in the levels of induction of these genes between donors, such as up to 200 000 times for Donor 59 and up to 1500 for Donor 76 for *COL2A1* mRNA relative expression compared to undifferentiated hMSCs (S4 Fig), the results were not statistically significant. However, when analysing these in donors individually (S4 Fig), the decrease of *COL2A1*, *ACAN*, and *SOX9* relative mRNA expression after irradiation was statistically significant. A trend towards an increase in *RUNX2* levels was also observed after irradiation, with the mean mRNA expression levels going from 1.4 to 2.2 (x1.5). This increase was statistically significant when analysing donors individually (S4 Fig), and suggests that osteogenesis might be favoured after irradiation, to the detriment of chondrogenesis.

More specifically, the decreased mRNA relative expression of *COL2A1*, *ACAN* and *SOX9* after irradiation, suggests that X-rays irradiation affects the ability of hMSCs to differentiate into chondrocytes.

### Collagen expression by chondrogenically differentiated hMSCs is impaired after irradiation

We then looked at the expression of type II collagen, a marker of chondrocytes, and that of type I collagen, a marker of dedifferentiated chondrocytes and osteoblasts, in chondrogenically differentiated chondrocytes after irradiation. As seen in Fig 4D, culture of hMSCs in 3D and in physioxia (CH 3D-hMSCs-0 Gy) in the presence of the chondrogenic medium resulted in an enhanced expression of the procollagen and the mature form of type II collagen in comparison to non-differentiated hMSCs (ND-hMSCs-0 Gy). The level of type II collagen expression in the differentiated chondrocytes was comparable to that of human articular chondrocytes (HAC), thus confirming induction of a chondrocytic phenotype in these culture conditions. A slight increase in the expression of both the procollagen and the mature form of type I collagen was also observed in the differentiated hMSCs *versus* the non-differentiated hMSCs. After irradiation, a slight decrease in the mature form of type I collagen was observed, while a decrease in both the procollagen and the mature form of type II collagen was observed, thus confirming impairment of the chondrogenesis differentiation process.

The decreased expression of type II collagen (Fig 4D) was associated not only with the trend towards a decreased *COL2A1* mRNA expression (Fig 4C), but also with an increase in *MMP-13* mRNA expression, a marker of hypertrophic chondrocytes (Fig 4E). When expression of *MMP-3* and *MMP-13* were analysed in individual donors, the increase of *MMP-3*, and *MMP-13* was statistically significant between the non-differentiated hMSCs (ND-hMSCs) and the chondrogenically differentiated hMSCs (CH 3D-hMSCs) (S3 Fig). The increase in *MMP-3* and *MMP-13* could contribute to the decreased expression of type II after X-irradiation.

## Discussion

Data presented here show that hMSCs are more radiosensitive than chondrosarcoma cells and that their radiosensitivity is not affected by culture in 3% O_2_. In addition, after a G2/M arrest both in normoxia and physioxia, hMSCs are able to resume proliferation. Finally, irradiation of hMSCs induces an impairment of both osteogenesis and chondrogenesis, characterised by an alteration of the collagen matrix composition.

Unlike the so-called radiosensitive tissues, for which early and late irradiation effects have been characterised at the cellular and molecular level [7], very few studies have investigated the effects of radiation on hMSCs [11,12,13,14,15,16]. We [13] and others [11,14,15,16] had previously shown that hMSCs were relatively radioresistant. However, it is well established that the responses of differentiated human cells to irradiation depend to a large extent on the physical characteristics of ionising radiations, such as the LET, the dose, the dose-rate, but also the biological characteristics of the cells at the time of irradiation, such as the proliferative state of the cells and their microenvironment, including O_2_ tension. Thus, in the present study, we used a medical linear accelerator for X-irradiation, we seeded hMSCs at a density that allowed them to be irradiated in the exponential phase, but also allowed a 16h delay for repair of DSBs, before replating them to assess survival [13,28]. Finally, in order to assess if the local oxygen microenvironment affects hMSCs responses to X-rays, we integrated the microenvironment by growing hMSCs in two different O_2_ environments, in normoxia (21% O_2_) and in physioxia (3% O_2_).

Radiosensitivity and cell cycle progression were thus studied in hMSCs grown in normoxia, which is the regular culture condition in laboratories, and in physioxia, that represents a more physiological environment for bone marrow stem cells [23]. In the context of bone tumours treatment by irradiation, including chondrosarcoma and osteosarcoma, hMSCs in the bone marrow or in the microenvironment of the tumours could be targets of irradiation [3]. We thus compared the sensitivity of hMSCs to chondrosarcoma cells, known to be radioresistant cancer cells [45]. This model is relevant when considering the responses of a healthy tissue in close proximity with a tumour. Our study shows that hMSCs were more radiosensitive than chondrosarcoma cells, and that their sensitivity was not affected by growing the cells in physioxia and normoxia. The relative radioresistance of hMSCs is in accordance with previous studies despite differences in the methods used to assess survival [11,13,14,15,16]. However, this is the first study to assess viability after 14 days in physioxia, and to show that hMSC viability in physioxia is comparable to that in normoxia. The biological effects of irradiation are largely mediated by reactive oxygen species whose production is disturbed in a hypoxic environment. Since physioxia is thus known to provide radioprotection, one could have expected hMSC survival in normoxia to be lower than in physioxia. The absence of differences in hMSC survival in normoxia and in physioxia could be explained by the fact that hMSCs within the body are in a rather hypoxic environment, and have thus developed efficient signalling processes to deal with irradiation insults even in a normoxic environment. One example of this is the high expression of HIF-1α, a transcription factor activated in response to physioxia, in hMSCs grown in normoxia [46], giving them the same level of radioprotection in normoxia as in physioxia. hMSCs are also known to be equipped with high antioxidant activities, which are likely to promote resistance to irradiation-induced oxidative stress even in a normoxic environment [11].

These explanations are also relevant to our results in cell cycle progression, showing no difference in the cell cycle progression after irradiation between hMSCs grown in normoxia and hMSCs grown in physioxia. It is known that irradiation induces DSBs, to which cells respond by activating the DDR, leading mainly to a G2/M arrest and ultimately cell death in a variety of cells [18]. Cell cycle progression of hMSCs was indeed influenced after irradiation, with a decreased number of cells in S phase and a remarkable increase of cells in G2/M indicative of an irradiation-induced G2/M arrest, both in normoxia and in physioxia. A higher number of cells in G2/M was still observed in irradiated cells compared to non-irradiated cells 7 days after irradiation, but lower than 16h after irradiation. Seven days after irradiation, an increase in the number of cells in S phase was also observed compared to the number of cells in S phase 16h after irradiation. These results are indicative of a G2/M arrest at or prior to 16h and a release from the G2/M arrest, at least in a subset of the hMSCs population, leading to hMSCs progressing through the cell cycle 7 days after irradiation, both in normoxia and physioxia, even it is not back to normal status.

Cellular morphology could have been also influenced by irradiation, however, no major differences between normoxia and physioxia conditions and no evidence of apoptosis were detected. This, together with the absence of an increase in subG1 after irradiation in normoxia and in physioxia and the presence of senescent cells after 10 Gy both in normoxia and in physioxia, suggest that, as previously described, hMSCs undergo radiation-induced premature senescence rather than apoptosis [11,12,19,47]. Avoidance of radiation-induced cell death by hMSCs is probably due to efficient activation of the DDR pathway, leading to cell cycle arrest to allow time for DNA damage repair [3,17]. hMSCs have indeed been shown to respond to irradiation by activating the DDR through increase in *PCNA*, *ATM* or *CHK2* [15], but more specifically ATM and DNA-PK phosphorylation and DSB repair probably through the non-homologous end-joining repair mechanism [11,13,20]. High antioxidant capacities of hMSCs have also been implicated in their relative radioresistance [11]. All together our results are in agreement with previous studies [11,13,15] showing that hMSCs were still able to proliferate after irradiation. However, it is the first study integrating the O_2_ tension [23] and showing that this does not influence hMSCs cycle progression in response to irradiation.

The response of adult stem cells to ionizing radiation must be a fine balance between maintenance of tissue homeostasis and genomic integrity. The choice of self-renewal, differentiation or death is critical in determining stem cell number. For instance massive elimination of damaged cells is efficient to decrease the risk of cell transformation, but can compromise lineage functionality. On the other hand, radiation resistance resulting in survival of cells with unrepaired damage can favour long-term accumulation of genetic anomalies [7]. Study by Kurpinsky *et al*. [14] presented different transcriptomic responses between ^56^Fe ions (high LET) and X-rays (low LET) treatments at the level of genes involved in cell cycle regulation, DNA replication and proliferation, thus suggesting radiation-induced modifications of the proliferation/differentiation balance in hMSCs. Alterations in the process of differentiation may be detrimental to the balance within the bone marrow and for the mobilisation of hMSCs in response to damage, and may participate to cancer induction [48,49]. Our results suggest that hMSCs favour tissue homeostasis in response to irradiation, since hMSCs are able to survive and proliferate after irradiation. Their role in maintaining tissue homeostasis relies in their capacities to undergo differentiation into specialised cell types, which are adipocytes, chondrocytes and osteoblasts, in order to give rise to different tissues, including adipose tissue, cartilage and bone [1]. However, data on the effects of irradiation on hMSCs differentiation capacities are very limited [11,15,16,50], and still remain controversial [3,17]. Differences in the results might come from differences in cell origin (bone marrow or umbilical cord), irradiation conditions (cells in suspension, seeding densities, LET, doses, dose-rate) and experimental designs, with differentiation being induced immediately [16], 6h [50] and up to 5 days after irradiation [11]. Considering the role of hMSCs in both bone formation and cartilage formation through osteogenesis and chondrogenesis [51], we focused on the radiation-induced effects on the osteogenic and chondrogenic differentiation abilities of hMSCs.

Osteogenic differentiation was induced using conditions known to be favourable for osteogenic differentiation [31,33]. The osteogenic differentiation process was induced 16h after irradiation, where hMSCs are in G2/M (Fig 2) and DSBs repaired γ-H2AX gone anyway (S1 Fig and [13]). Detailed analysis of osteogenic differentiation after irradiation showed that osteogenic differentiation is increased after irradiation as seen by an increase in calcium deposition and an increase in relative mRNA expression of specific osteogenic genes such as alkaline phosphatase (*ALP*) and osteopontin (*OPN*) (osteogenic markers [38]). Osteogenic differentiation, despite increasing after irradiation, may however be altered since matrix deposition seemed impaired as seen by decreased expression of one osteoblasts extracellular matrix protein, type I collagen. In the same vein, this impaired matrix deposition seems to be confirmed when analysing the expression of the late marker of the mature osteoblasts, *BSP*, which is increased. Another late marker of osteogenesis, *OCN*, is however not modulated, neither after differentiation induction nor after irradiation. The absence of OCN modulation might be inked to the use of dexamethasone [33] over the use of 1,25-dihydroxyvitamin D3 [52] in the differentiation medium. Nevertheless, the increase in *ALP*, *OPN*, and *BSP*, are in favour of an alteration of osteogenesis differentiation after irradiation. The differentiation of hMSCs into osteoblasts is a complex and highly regulated process defined by four consecutive stages, lineage commitment, proliferation, extracellular matrix maturation and matrix mineralization. Type I collagen is of importance for proper bone matrix organisation and matrix mineralization during the development of the bone. Abnormalities in synthesis or processing of type I collagen have been involved in bone diseases such as osteoporosis, osteoarthritis, rheumatoid arthritis and *osteogenesis imperfecta* [38]. Since our real-time PCR results show that type I and type II collagen synthesis is not impaired, a possible explanation for the observed decreased expression of the proteins relies in the degradation of the collagens by matrix metalloproteinase (MMP) family members. MMP-1 (collagenase-1), MMP-3 (stromelysin) and MMP-13 (collagenase-3) are members of the MMP family known to degrade components of the extracellular matrix. MMP-1 and MMP-3 are among the most ubiquitously expressed MMPs, and play a major role in the degradation of the interstitial collagens (types I, II and III). MMP-13 is usually produced only by cartilage and bone during development, and by chondrocytes in osteoarthritis, and hydrolyzes type II collagen more efficiently than the other collagenases [53]. Our results show that *MMP-3* relative mRNA expression is increased after irradiation in osteogenically differentiated hMSCs. This increase could contribute to the decreased expression of type I collagen observed after irradiation in osteogenically differentiated hMSCs. The collagen fibres comprise about 90% of the total protein in the bone, and decrease/degradation in type I collagen, as observed here after irradiation might result in abnormal extracellular matrix structure and/or properties and ultimately in bone defects [38,54,55]. Since the post-yield properties of bone (strength, toughness) are thought to rely highly on an intact collagen network [55], such alteration could affect strength and toughness of the bone formed after irradiation. Moreover, growth dissymmetry in children after conventional RT is well-known, and could be explained in part by radiation-induced defects in osteogenic differentiation of hMSCs and by osteogenic matrix degradation as observed in our study, as well as radiation-induced cell death of preosteoblasts [20,56]. Very few studies reported the effects of irradiation on osteogenesis differentiation of hMSCs [3,11,15,16,50]. Studies by Nicolay *et al*, [15] and Chen *et al* [11] suggested that hMSCs retain their osteogenic differentiation abilities after irradiation. However, both these studies focused mostly on qualitative determination of osteogenesis (ie calcium deposition). A stable expression of *BMP-6* and *RUNX2* after irradiation was though reported by Nicolay *et al*, in favour of no alteration of osteogenesis [15]. However, since only *BMP-6* is the specific osteogenesis marker studied, it is difficult to strictly conclude that osteogenesis is not altered. On the opposite, Hou *et al* [50] and Li *et al*, [16] reported that osteogenic differentiation potential of hMSCs was damaged by irradiation. These two studies provided analysis of osteogenic related markers, including *ALP* [16,50] and *OGN* [50] both being decreased after irradiation. These results are in agreement with an alteration of osteogenesis after irradiation. However, they showed a decrease in *ALP* after irradiation, while we saw an increase. This discrepancy might be linked to experimental variations. Irradiation in suspension, followed by replating at low density and induction of differentiation induced 24h later [16], might not allow appropriate confluency for osteogenesis induction. Osteogenesis analysis at day 14, instead of day 21, and induction of osteogenesis only 6h after irradiation [50], might not allow cells to repair damage. Our study is thus the first study showing impairment of osteogenesis after irradiation, through both qualitative analysis, and detailed analysis of osteogenesis differentiation-related genes, transcription factors, and extracellular matrix proteins.

The process of chondrogenesis has been the subject of numerous studies in the field of tissue engineering for the treatment of cartilage defects [57]. Studies firstly focused on phenotypically stabilizing chondrocytes in culture but are currently focusing on the use of progenitor stem cells. Chondrogenesis can indeed be induced in hMSCs using favourable culture conditions, such as a three-dimensional culture format, low oxygen tension, and the presence of appropriate growth factors, such as members of the TGF-β family as well as bone morphogenic proteins [30,39,40]. We first induced chondrogenesis 16h after irradiation in a two-dimensional culture format, using TGF-β1 and BMP-2 as chondrogenesis inducers, in order to analyse the effects of irradiation on chondrogenesis in comparable conditions as for osteogenesis. When looking at chondrocytes specific markers (*COL2A1*, *SOX9*, *ACAN*), only a significant increase in the relative mRNA expression of *SOX9* was observed when hMSCs were grown in the chondrogenic medium. However, an increase in the relative mRNA expression of *COL1A1*, *COL10A1*, and *RUNX2*, markers of dedifferentiated and hypertrophic chondrocytes, and pre-osteoblasts, was also observed, indicating poor chondrogenesis. This was expected as a three dimensional culture format and low oxygen tension have been shown to be important for proper *in vitro* chondrogenesis [30]. However, when looking at the effect of irradiation on chondrogenesis differentiation in these conditions, the decrease in *ACAN* and *SOX9* relative mRNA expression associated with an increase in *COL1A1*, *COL10A1* and *RUNX2*, suggested an alteration of the chondrogenic differentiation ability of hMSCs, towards hypertrophic chondrocytes. Since the conditions were not ideal in inducing chondrogenesis, we took advantage of our experience with human articular chondrocytes redifferentiation model [26,27,32], and applied it to hMSCs. This model integrates all favourable conditions to induce proper chondrogenesis, *ie* a three dimensional culture format, low oxygen tension, and appropriate growth factors. Chondrogenesis was thus induced in hMSCs included in type I/III collagen sponges [26,58], in media containing TGF-β1, and BMP-2 and in 3% O_2_ [26]. The highly elevated relative mRNA expression of *COL2A1*, *ACAN* and *SOX9*, with limited increase in *COL1A1*, and the high expression of type II collagen, validated the use of this model to induce chondrogenesis.

When looking at the radiation-induced effects on chondrogenesis in this system, the decrease of the relative mRNA expression of specific markers of chondrocytes (*COL2A1*, *ACAN*, *SOX9*), associated with an increase of the relative mRNA expression of the hypertrophic and osteoblastic marker *RUNX2*, suggested that chondrogenesis was altered. Alteration of chondrogenesis did not seem to be in favour of chondrocytes full hypertrophy maturation since no increase in neither *COL1A1* and *COL10A1* relative mRNA expression nor in type I collagen was observed [40]. The increase in *RUNX2* though suggests that the phenotype favoured might be that of osteoblasts. However, again no increase in type I collagen was observed, thus suggesting that even if an osteoblastic phenotype might be favoured it would present an incorrect matrix composition. In fact a decrease in both *COL2A1* relative mRNA expression and in type II collagen expression was observed after irradiation, thus confirming a decrease in synthesis of this chondrocyte specific protein. Our results also show that the relative mRNA expression of *MMP-13*, a very potent type II collagen proteinase [53] and a hypertrophy marker is increased after irradiation in chondrogenically differentiated hMSCs. Thus the decrease in type II collagen expression after irradiation in chondrogenically differentiated hMSCs might not only result from a decrease in synthesis but also an increase in type II collagen degradation by MMP-13. The very few studies [11,15,16,47], reporting the effects of irradiation on differentiation of hMSCs only dealt with the effects of irradiation on adipogenesis and ostegenesis, probably due to the difficulties in inducing and maintaining proper chondrogenesis *in vitro* [40]. This is thus the first study reporting the effects of irradiation on hMSCs chondrogenesis in conditions mimicking the natural hMSCs microenvironment.

Taken together our results show that irradiation of hMSCs induces an impairment of both osteogenesis and chondrogenesis, characterised by an alteration of the matrix composition formed by the osteoblasts and chondrocytes. Decreased expression of both type I and II collagens during osteogenesis after irradiation may mostly reflect increased degradation of these proteins by MMP-3 and MMP-13. In the case of decreased expression of type II collagen during the process of chondrogenesis after irradiation, an inhibition of type II collagen synthesis associated with an increase in type II collagen degradation by MMP-13 can explain the decreased expression observed for articular cartilage [59]. Consequences of matrix composition defects during the process of hMSCs differentiation towards osteoblasts and chondrocytes might include alteration of bone formation and bone toughness, as well as alteration of endochondral ossification, loss of chondral substance and loss of joint function. The radiation doses provided to bone and cartilage during conventional RT is minimized in order to reduce the incidence of bone damage and growth abnormalities. However, both fractures, joint deterioration and growth dissymmetry are known radiation-induced long term sequelae [60]. Growth dissymmetry observed in children after conventional RT, might be explained by the direct effects of irradiation on bone and chondrocytes [59]. However, it is conceivable from our present data that alteration of the osteogenic and chondrogenic differentiation of their progenitors, the mesenchymal stem cells, might also account for the subsequent abnormal bone and skeletal growth observed after radiation therapy.

## Supporting Information

S1 Fig Western blot analysis of γ-H2AX, 1h and 16h following X-irradiation. Cells grown in normoxia for 48h before X-rays irradiation, were harvested for western blotting 1h and 16h following X-rays irradiation. Western blot analysis of γ-H2AX and p53 was performed as described in Material and Methods.
(TIF)

S2 Fig Real-time RT-PCR analysis of osteogenesis markers in hMSCs differentiated into osteoblasts and obtained from two independent donors (A-Donor 61 and B-Donor 76). Real-time RT-PCR analysis of relative mRNA expression of the indicated genes (ALP-alkaline phosphatase, *OPN*-Osteopontin, *BSP*-Bone sialoprotein) in undifferentiated hMSCs (ND-hMSCs) and osteogenically-differentiated hMSCs (OS-hMSCs) after irradiation (0-6-10 Gy). Gene expression is normalized against the endogenous reference gene, *RPL13A*. Results are expressed as relative mRNA expression as compared to the control condition (undifferentiated hMSCs, 0 Gy condition) set at the arbitrary unit of 1. Column bar graphs (Mean ± SEM) of 3 wells. Statistical analysis (unpaired Student t test) was performed and statistical significance is shown with, * for p<0.05, ** for p< 0.01, *** for p<0.001
(TIF)

S3 Fig Real-time RT-PCR analysis of MMPs in hMSCs differentiated into osteoblasts or into chondrocytes in 3D and physioxia, and obtained from two independent donors (Donor 59 for *MMP-3* and Donor 61 for *MMP-13*). Real-time RT-PCR analysis of relative mRNA expression of the indicated genes (*MMP-3*-matrix metalloproteinase 3, *MMP-13* matrix metalloproteinase 13) in undifferentiated hMSCs (hMSCs-ND) and either osteogenically differentiated (A) OS-hMSCs) or chondrogenically differentiated hMSCs (B) CH 3D-hMSCs) after irradiation (0-6-10 Gy). Gene expression is normalized against the endogenous reference gene, *RPL13A*. Results are expressed as relative mRNA expression as compared to the control condition (undifferentiated hMSCs 0Gy condition) set at the arbitrary unit of 1. Column bar graphs (Mean ± SEM) of 3 wells. Statistical analysis (unpaired Student t test) was performed and statistical significance is shown with, * for p<0.05, ** for p< 0.01, *** for p<0.001.
(TIF)

S4 Fig Real-time RT-PCR analysis of chondrogenesis markers in hMSCs differentiated into chondrocytes in 3D and physioxia, and obtained from two independent donors (A-Donor 59 and B-Donor 76). Real-time RT-PCR analysis of relative mRNA expression of the indicated genes (*COL2A1*-type II collagen, *ACAN*-aggrecan, *SOX9*-Sox9 transcription factor, *RUNX2*-runt-related transcription factor 2) in undifferentiated hMSCs (ND-hMSCs) and chondrogenically differentiated hMSCs (CH 3D-hMSCs-) after irradiation (0-6-10 Gy). Gene expression is normalized against the endogenous reference gene, *RPL13A*. Results are expressed as relative mRNA expression as compared to the control condition (undifferentiated hMSCs 0Gy condition) set at the arbitrary unit of 1. Column bar graphs (Mean ± SEM) of 3 wells. Statistical analysis (unpaired Student t test) was performed and statistical significance is shown with, * for p<0.05, ** for p< 0.01, *** for p<0.001.
(TIF)
