# Supplementary figures and images for: Radiation-Induced Alterations of Osteogenic and Chondrogenic Differentiation of Human Mesenchymal Stem Cells
Source: PLoS One. 2015 Apr 2;10(4):e0119334. doi: 10.1371/journal.pone.0119334 (PMC4383487; doi:10.1371/journal.pone.0119334)

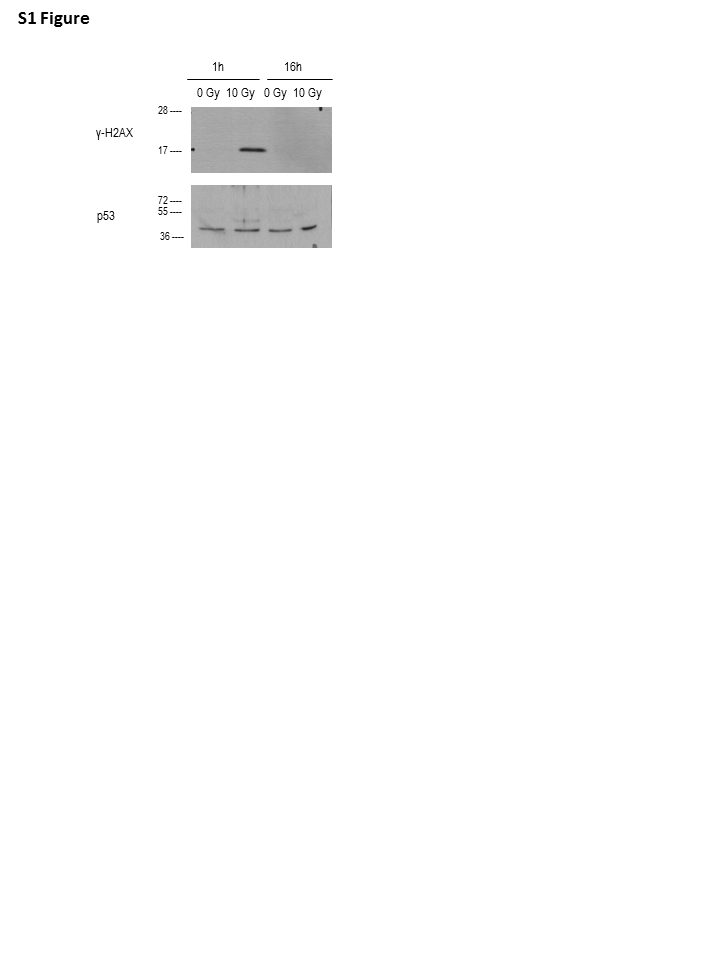

Supplement: S1 Fig — Cells grown in normoxia for 48h before X-rays irradiation, were harvested for western blotting 1h and 16h following X-rays irradiation. Western blot analysis of γ-H2AX and p53 was performed as described in Material and Methods. (TIF) [file pone.0119334.s001.tif]

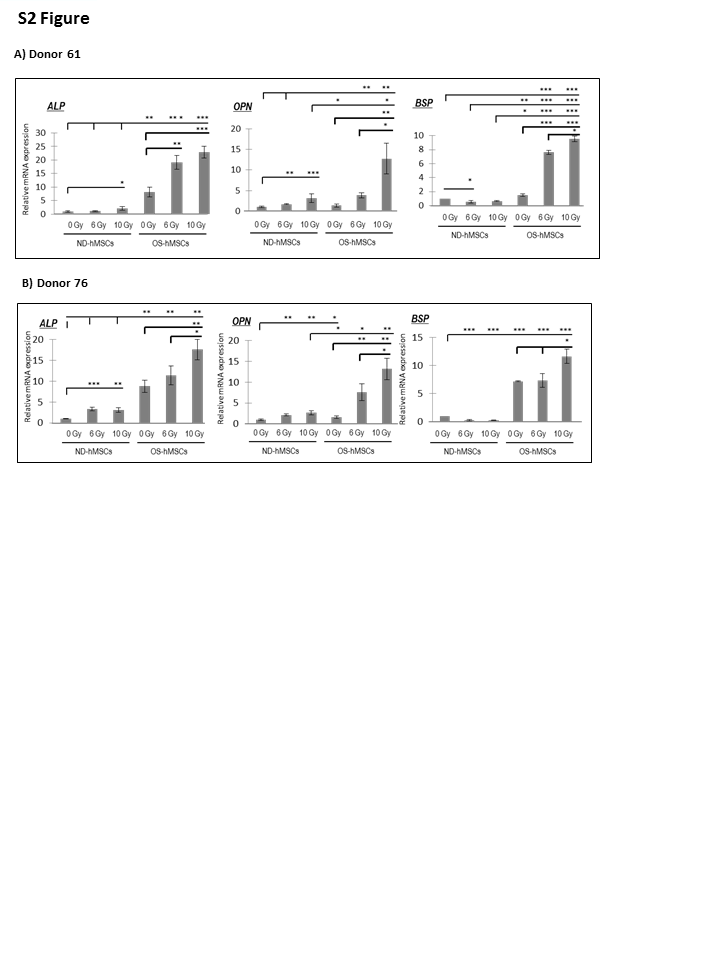

Supplement: S2 Fig — Real-time RT-PCR analysis of relative mRNA expression of the indicated genes (ALP-alkaline phosphatase, OPN-Osteopontin, BSP-Bone sialoprotein) in undifferentiated hMSCs (ND-hMSCs) and osteogenically-differentiated hMSCs (OS-hMSCs) after irradiation (0-6-10 Gy). Gene expression is normalized against the endogenous reference gene, RPL13A. Results are expressed as relative mRNA expression as compared to the control condition (undifferentiated hMSCs, 0 Gy condition) set at the arbitrary unit of 1. Column bar graphs (Mean ± SEM) of 3 wells. Statistical analysis (unpaired Student t test) was performed and statistical significance is shown with, * for p<0.05, ** for p< 0.01, *** for p<0.001 (TIF) [file pone.0119334.s002.tif]

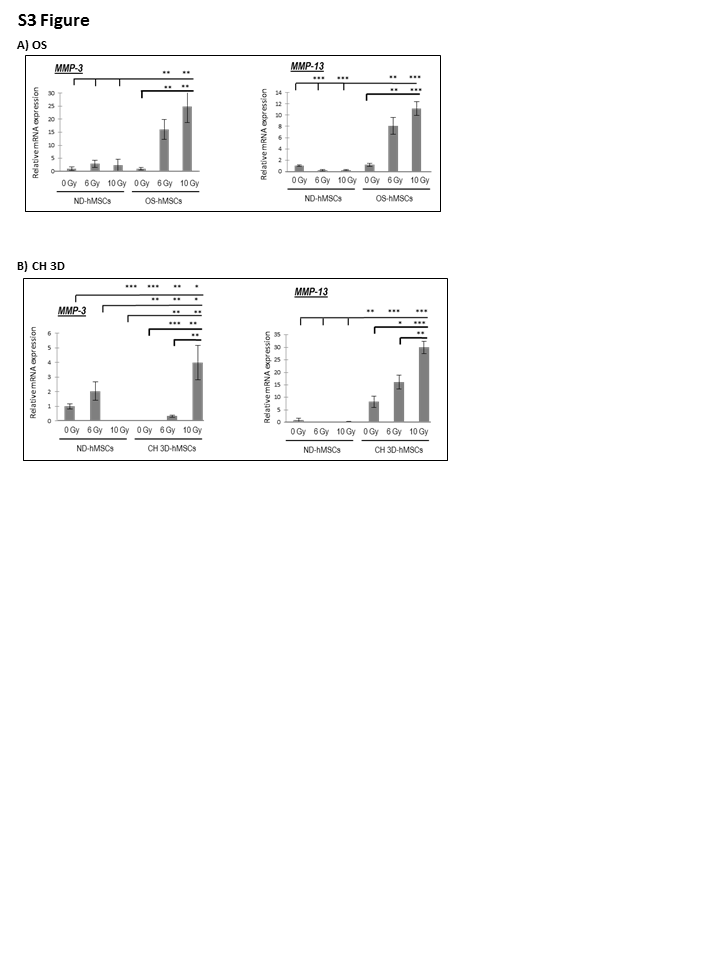

Supplement: S3 Fig — Real-time RT-PCR analysis of relative mRNA expression of the indicated genes (MMP-3-matrix metalloproteinase 3, MMP-13 matrix metalloproteinase 13) in undifferentiated hMSCs (hMSCs-ND) and either osteogenically differentiated (A) OS-hMSCs) or chondrogenically differentiated hMSCs (B) CH 3D-hMSCs) after irradiation (0-6-10 Gy). Gene expression is normalized against the endogenous reference gene, RPL13A. Results are expressed as relative mRNA expression as compared to the control condition (undifferentiated hMSCs 0Gy condition) set at the arbitrary unit of 1. Column bar graphs (Mean ± SEM) of 3 wells. Statistical analysis (unpaired Student t test) was performed and statistical significance is shown with, * for p<0.05, ** for p< 0.01, *** for p<0.001. (TIF) [file pone.0119334.s003.tif]

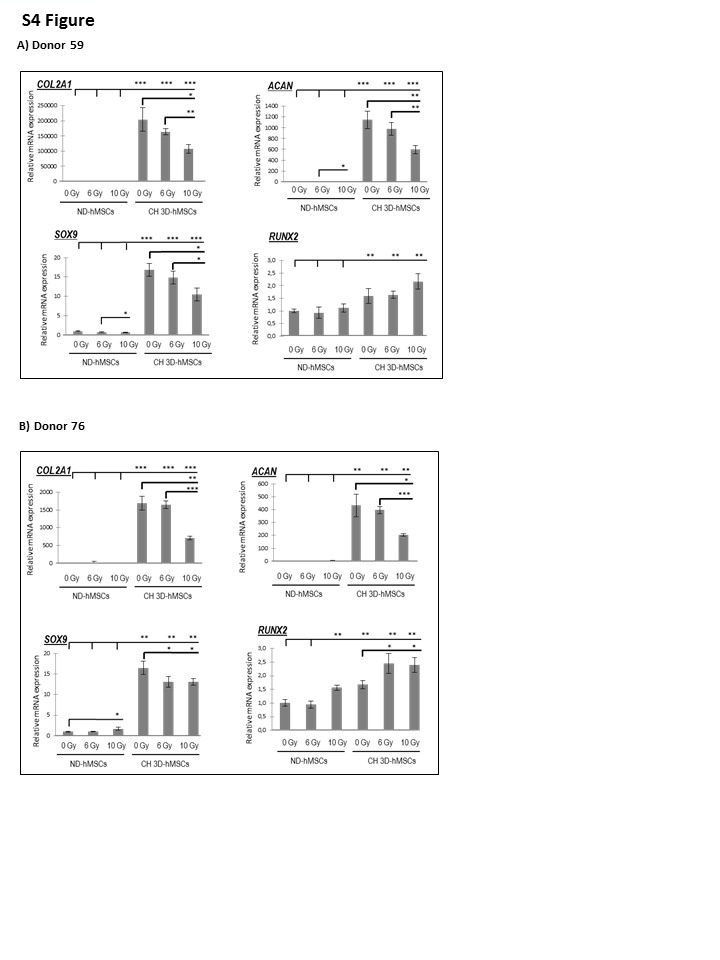

Supplement: S4 Fig — Real-time RT-PCR analysis of relative mRNA expression of the indicated genes (COL2A1-type II collagen, ACAN-aggrecan, SOX9-Sox9 transcription factor, RUNX2-runt-related transcription factor 2) in undifferentiated hMSCs (ND-hMSCs) and chondrogenically differentiated hMSCs (CH 3D-hMSCs-) after irradiation (0-6-10 Gy). Gene expression is normalized against the endogenous reference gene, RPL13A. Results are expressed as relative mRNA expression as compared to the control condition (undifferentiated hMSCs 0Gy condition) set at the arbitrary unit of 1. Column bar graphs (Mean ± SEM) of 3 wells. Statistical analysis (unpaired Student t test) was performed and statistical significance is shown with, * for p<0.05, ** for p< 0.01, *** for p<0.001. (TIF) [file pone.0119334.s004.tif]
